# Fish Oil Monotherapy for Intestinal Failure-Associated Liver Disease on SMOFlipid in the Neonatal Intensive Care Unit

**DOI:** 10.3390/jcm9113393

**Published:** 2020-10-23

**Authors:** Sanghoon Lee, Se In Sung, Hyo Jung Park, Yun Sil Chang, Won Soon Park, Jeong-Meen Seo

**Affiliations:** 1 Intestinal Rehabilitation Team, 81 Irwon-ro, Gangnam-gu, Seoul 06351, Korea; 4hooni@gmail.com (S.L.); hyoj.park@samsung.com (H.J.P.); 2 Department of Surgery, Samsung Medical Center, Sungkyunkwan University School of Medicine, 81 Irwon-ro, Gangnam-gu, Seoul 06351, Korea; 3 Department of Pediatrics and Adolescents, 81 Irwon-ro, Gangnam-gu, Seoul 06351, Korea; sein.sung@samsung.com (S.I.S.); cys.chang@samsung.com (Y.S.C.); ws123.park@samsung.com (W.S.P.); 4 Department of Pharmaceutical Services, Samsung Medical Center, Sungkyunkwan University School of Medicine, Seoul 06351, Korea

**Keywords:** intestinal failure-associated liver disease, parenteral nutrition, intravenous lipid emulsion, fish oil, premature, infant

## Abstract

Intestinal failure-associated liver disease (IFALD) is a life-threatening complication of parenteral nutrition (PN) and is most prevalent in the preterm neonatal population receiving long-term PN. In this study, we report the outcome of our experience with fish oil monotherapy for IFALD in a fish oil-based combination lipid emulsion administered to preterm low birth weight infants. Fasting neonates were administered as PN according to our center’s nutrition protocol. A diagnosis of IFALD was made when the serum direct bilirubin levels were >2.0 mg/dL in two consecutive measurements that were more than one week apart, without evidence of intrinsic causes of liver dysfunction. The management of IFALD was conducted by switching the lipid emulsion from combination lipid emulsion to fish oil monotherapy at 1.0 g/kg/day, infused over 24 h. Fifteen infants met the criteria for IFALD and received fish oil monotherapy. The median gestational age was 27.5 weeks and the median birth weight was 862.5 g. IFALD was successfully reversed in 11 infants (11/15, 73.3%). The median duration of fish oil monotherapy was 39 days. Direct bilirubin values were initially elevated and then steadily declined from the third week of treatment onward. The enteral tolerance increased in varying degrees during the treatment period. The mean weight gain was 26.0 g/day during fish oil monotherapy. Omegaven^®^ (Fresenius Kabi Austria Gmbh, Graz, Austria) at a dose of 1.0 g/kg/day was well tolerated, and no adverse events related to Omegaven use were seen. The reversal of IFALD in preterm infants on combination lipid emulsion containing fish oil was achieved by switching to fish oil monotherapy.

## 1. Introduction

The theoretical background for using combination lipid emulsions (LE) containing fish oil is based upon utilizing the advantages of each oil source (i.e., essential fatty acids in soybean oil, anti-inflammatory properties of fish oil) while reducing the disadvantages of relying on a sole source of oil (i.e., phytosterols in soybean oil) [1]. SMOFlipid 20% (SMOF; Fresenius Kabi Austria Gmbh, Austria) is a combination intravenous LE composed of four lipid sources: soybean oil, medium-chain triglycerides (MCTs), olive oil, and fish oil (FO) in a 6:6:5:3 ratio. SMOF was approved by the US Food and Drug Administration in 2016 for use in adults. It has been approved and available for use in both children and adult patients in Korea and many centers, including ours, have been using SMOF as the LE of choice since 2008.

The clinical advantage of using combination LEs such as SMOF is most relevant in the preservation of liver function during long-term parenteral nutrition (PN) and has been shown to be beneficial in this aspect by previous reports [2,3]. Intestinal failure-associated liver disease (IFALD) is a life-threatening complication of PN and is most prevalent in the preterm neonatal population receiving long-term PN. The pathogenesis of IFALD is multifactorial, while the amount and content of the LE used is closely related to IFALD development and progression.

The management of IFALD using FO monotherapy with Omegaven (Fresenius Kabi Austria Gmbh, Austria) has repeatedly been shown to be safe and effective in children and, more recently, also in preterm neonates who are on long-term PN [4]. However, all of these studies have dealt with IFALD occurring while using soybean oil-based LE. The question arises of whether the same approach of soybean oil elimination and the sole use of FO would also be feasible when IFALD occurs while using fish oil-based LE in the PN regimen, as is the case with SMOF. We have previously reported two cases in which IFALD in infants on long-term PN composed of SMOF were completely reversed with conventional FO monotherapy [5]. In the present study, we report the outcome of our expanded experience with FO monotherapy for IFALD on fish oil-based combination LE in the pediatric population, particularly in preterm low birth weight infants.

## 2. Materials and Methods

### 2.1. Study Population

This study is a retrospective analysis of patients who were admitted in the neonatal intensive care unit (NICU) at Samsung Medical Center (Seoul, Korea) from March 2017 to June 2018. Patients were included if they developed IFALD while on PN support and received FO monotherapy for the management of IFALD.

A historical cohort of infants managed in our NICU in the era before the application of FO monotherapy for IFALD was selected by a retrospective review of medical records. Patients were included if they developed IFALD while on PN support and received lipid reduction therapy (LE reduced to 1 g/kg/day of SMOF) from October 2012 to February 2017.

### 2.2. Parenteral and Enteral Nutrition Support Protocol

Fasting neonates were administered in PN according to our center’s nutrition protocol, which consisted of carbohydrates (15~18 g/kg/day), amino acids (3.0~3.5 g/kg/day), and lipids as SMOF at 2.0~3.0 g/kg/day over 24 h. Appropriate amounts of electrolytes, micronutrients, and vitamins were also included. All the PN prescriptions were individualized to meet each infant’s needs and prepared on a daily basis.

Blood samples were analyzed weekly to biweekly and included the complete blood count, electrolytes, liver enzymes, bilirubin, protein, serum albumin, and C-reactive protein, as clinically indicated. A diagnosis of IFALD was made when the serum direct bilirubin levels were >2.0 mg/dL in two consecutive measurements that were more than one week apart, without the presence of conditions which may contribute to cholestasis and liver dysfunction such as viral hepatitis, metabolic liver disease, structural anomalies of the hepatobiliary system, ongoing infection or sepsis, prolonged antibiotics therapy, and congenital heart diseases. Conversely, the reversal of IFALD was considered to have occurred when the serum direct bilirubin levels were ≤2.0 mg/dL in two consecutive measurements that were more than one week apart.

Enteral intake was advocated in all instances, as tolerated by the patient. The tolerance to enteral intake was assessed daily by the attending physician. Abdominal distension and softness to touch and the amount and color of gastric residues were assessed.

All the patients’ daily managements were conducted by a team of neonatologists and pediatric surgeons. A multidisciplinary team consisting of pediatric surgeons, neonatologists, and pharmacists were also in charge of prescribing appropriate amounts of enteral feeds and PN. Patients were monitored daily for changes in vital signs, urine and stool output, and weight by the multidisciplinary team.

### 2.3. IFALD Management Protocol

The management of IFALD was carried out by initially decreasing the total calories provided by PN, usually by lowering the amount of lipid to 2.0 g/kg/day. When calorie decrement did not result in the alleviation of cholestasis, fish oil monotherapy was initiated by switching the LE from SMOF to Omegaven at 1.0 g/kg/day infused over 24 h. Other known measures attempting the management of IFALD such as PN cycling or ursodeoxycholic acid were not employed in our center’s nutrition support protocol. Fish oil monotherapy was discontinued when the patient achieved sufficient enteral tolerance enough to discontinue PN, along with intravenous LE or IFALD reversal being achieved. When the patient achieved IFALD reversal while still receiving PN support, LE was switched back to SMOF at 1.0 g/kg/day. With continuing stable liver function, SMOF was carefully increased to 2.0 g/kg/day and beyond after several days.

The management of IFALD was carried out by lipid reduction therapy prior to this study period at our NICU. Patients were given a reduced dose of lipids (LE reduced to 1 g/kg/day of SMOF), resulting in a reduction in the overall PN calories and calories provided by lipids.

### 2.4. Statistical Anaylsis

The patient characteristics and variables were compared using the Student’s *t*-test for continuous variables and Fisher’s exact test for categorical variables. Statistical significance was accepted at *p* < 0.05. Statistical analyses were performed using SAS, version 9.4 (SAS Institute, Cary, NC, USA).

This study was carried out in accordance with relevant guidelines and regulations. This study was approved by the Institutional Review Board at Samsung Medical Center (2020-04-173).

## 3. Results

Fifteen infants met the criteria for IFALD and received FO monotherapy during the study period from March 2017 to June 2018 (Table 1). The median gestational age was 27.5 weeks and the median birth weight was 862.5 g. All the patients required prolonged PN support following open laparotomy, with underlying pathologies including necrotizing enterocolitis in 8 cases, meconium-related ileus in 6 cases, and short bowel due to midgut volvulus in 1 case. IFALD was diagnosed and FO monotherapy was initiated at median 60 days of life (DOL). IFALD was successfully reversed in 11 infants (11/15, 73.3%). Four infants died of IFALD and resultant hepatic failure. One infant (case #9) was cleared of IFALD but later on suffered severe hypoxic brain injury and died. The median duration of FO monotherapy was 39 days.

**Figure 1:**
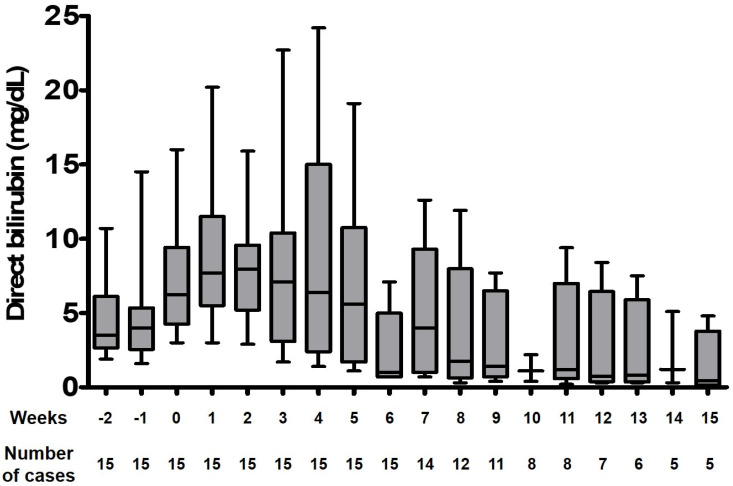
Direct bilirubin levels of 11 infants with intestinal failure-associated liver disease during fish oil monotherapy. Only data of those infants achieving IFALD reversal are represented in this graph. Week 0 indicates the time point of the initiation of fish oil monotherapy.

The direct bilirubin and total bilirubin levels at the beginning and end of FO monotherapy are shown in Table 2. Cases #6, #8, #11, and #14 progressed to hepatic failure and thus have elevated direct and total bilirubin at the end of treatment compared to the baseline values. Cases #1 and #3 show elevated direct and total bilirubin at the end of treatment, despite eventually having been cleared of IFALD—the two cases discontinued FO monotherapy as well as PN support itself because the infants were showing steady progress to full enteral tolerance. Cases #5, #7, and #9 displayed a clear downward trend in their direct bilirubin levels and opted to discontinue FO monotherapy and switch back to SMOF. The trends of direct bilirubin in infants with IFALD during FO monotherapy are shown in Figure 1. The median direct bilirubin values are initially elevated and then steadily decline from the third week of treatment onward. The additional biochemical parameters of liver function and triglyceride levels are also outlined in Table 2.

The enteral tolerance increased in varying degrees in all but one patient during the treatment period. The mean weight gain was 26.0 g/day during FO monotherapy. Omegaven at a dose of 1.0 g/kg/day was well tolerated in these infants during the entire period of administration, and no adverse events related to Omegaven use were seen. No signs of essential fatty acid deficiency (EFAD) were observed during FO monotherapy, although the fatty acid profiles were not measured.

A historical cohort of infants managed in our NICU in the era before the application of FO monotherapy for IFALD was selected by a retrospective review of medical records. From October 2012 to February 2017, 32 consecutive cases of infants met the criteria for IFALD and were managed with lipid reduction therapy (LE reduced to 1 g/kg/day of SMOF). The clinicopathologic characteristics and outcomes of two groups of infants with different IFALD management schemes are compared in Table 3. Patient survival was considerably higher in the FO monotherapy group, however the difference in survival was not statistically significant (*p* = 0.34).

## 4. Discussion

Preterm infants in the NICU are at high risk of feeding intolerance and often require PN support for prolonged periods of time. Lipids are an integral component of PN, and supplying adequate amount and composition of lipids is essential for growth, visual development, and cognitive development in the premature infant [6,7,8,9]. SMOF is a combination LE containing fish oil and is one of the fourth-generation LE products available [10]. SMOF has raised much interest among clinicians for its presumed anti-inflammatory properties and clinical benefits [11,12,13].

The use of combination LE containing fish oil in preterm infants has been subject to both hype and doubt due to its similarities in lipid composition to breast milk as well as concerns about insufficient essential fatty acids [1,4]. The safety and efficacy of combination LE in preterm infants have been argued in a study by Rayyan et al., in which the authors administered IV SMOF for 7 to 14 days in 26 neonates [3]. Adequate serum triglyceride levels were observed compared to the control group receiving soybean oil-based LE, and no serious adverse events related to the use of SMOF were documented. Moreover, the group of neonates receiving SMOF showed a significant decrease in direct bilirubin compared to controls. The authors concluded that SMOF was comparable to soybean-based LE in terms of providing a balanced fatty acid and adequate energy, promoting growth in the neonate, and was beneficial in reducing cholestasis. However, the added benefit of a reduction in cholestasis with FO in the PN regimen does not completely preclude the occurrence of IFALD. In fact, it has been documented that IFALD does occur, albeit less frequently, while using combination LEs such as SMOF [14,15]. In a previous study, we reported the incidence of IFALD to be 11.2% (28/251) among pediatric patients receiving PN containing SMOF for periods longer than 30 days and 12.3% in neonates [5]. However, there remains a gap in knowledge on the proper management and outcome of IFALD in this clinical situation.

Popular approaches to prevent and/or treat IFALD include PN cycling, maintaining oral or enteral intake (whenever possible), avoiding PN overfeeding, minimizing recurrent episodes of sepsis, and limiting the dose of soybean-based lipid to less than 1 g/kg/day [16]. However, the proportion of soybean-based lipid in SMOF is 30%, and less than 1 g/kg/day of soybean-based lipids would be given to a patient when SMOF is administered at 3 g/kg/day. Thus, the concept of (soybean-based) lipid reduction as a therapeutic approach would not apply in this situation, because the patient would already be in a state of “(soybean-based) lipid reduction” at 3 g/kg/day of SMOF. Moreover, concern has been raised with reducing SMOF to less than 2.0 g/kg/day due to the possibility of EFAD. Two recently published reports described five cases of documented EFAD occurring in infants receiving low doses (1.0 g/kg/day) of SMOF as a treatment strategy for IFALD [17,18]. Thus, an alternative therapeutic option for IFALD occurring on SMOF is called for, especially in this population of patients that are most susceptible to the consequences of EFAD.

In this study, we describe the outcome of FO monotherapy using 1.0 g/kg/day of Omegaven for IFALD occurring in infants on long-term PN with SMOF as their LE. Our results show trends of improved survival with FO monotherapy (11/15, 73.3%) compared to a historical cohort of infants managed only with lipid reduction prior to the FO era (18/32, 56.2%, *p* = 0.34). Additionally, similar rates of IFALD reversal were observed compared to previous reports of FO monotherapy for IFALD on soybean-based LE [19]. These results are consistent with our previous report of two cases and add to the still limited body of evidence concerning the treatment of IFALD occurring while using SMOF [5]. The safety and feasibility of FO monotherapy at a 1.0 g/kg/day dosage in preterm infants has been documented in a recent publication by Sorrell et al. [4]. No acute adverse events were observed in 13 preterm infants enrolled in the study, while the IFALD reversal rate was 100%. The growth (weight and head circumference) at 6 and 12 months of postmenstrual age was not different between the study population and matched controls. The group of infants in our study showed stable weight gain during the period of Omegaven monotherapy, from 1700 g at the initiation of Omegaven (37 weeks 3 days of gestation) to 2800 g at the discontinuation of Omegaven (43 weeks 4 days of gestation, all values are medians). This correlates to an improvement from less than 10 percentile range to a 10~25 percentile range of body weight [20].

**Figure 2:**
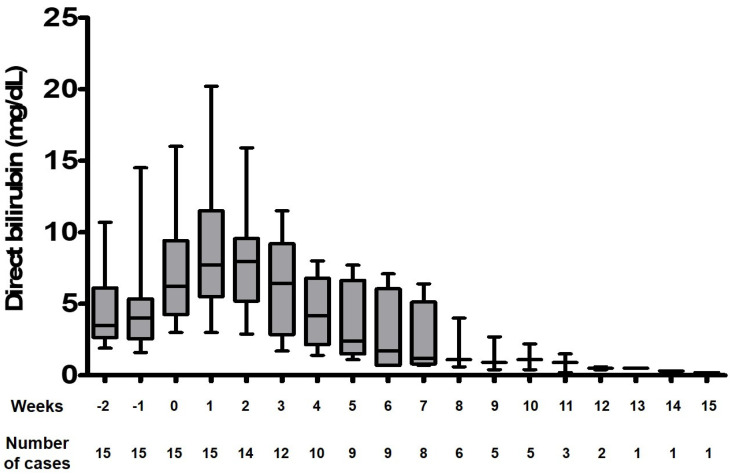
Direct bilirubin levels of 11 infants with intestinal failure-associated liver disease during fish oil monotherapy, during limited enteral intake. Data collected when the infant received less than 1/3 of required calories as enteral nutrition are represented in this graph. Week 0 indicates the time point of the initiation of fish oil monotherapy.

There are apparent limitations in the interpretation of our data, inherently due to the retrospective nature of this study. Most importantly, it may be contested that the resolution of cholestasis was more likely due to the increase in enteral intake and overall decrease in PN dependence. In fact, nearly all patients’ enteral tolerance increased from the time of initiation to the completion of FO monotherapy, albeit in varying degrees. However, the positive effect of Omegaven monotherapy is still apparent when the enteral intake factor is excluded, as seen in Figure 2; the resolution of cholestasis is consistently observed in patients with limited enteral tolerance (less than 1/3 of required calories given enterally). Another significant limitation is the lack of objective evidence that EFAD had not occurred in our infants. However, the prevention of EFAD in PN-dependent patients (including preterm infants) on FO monotherapy has been shown in numerous publications [21,22,23]. Finally, although we provide comparative analyses of the outcome of IFALD management between the study group and a historical cohort, the analyses are crude and statistically significant improvements in outcomes are lacking. This is probably due to the small number of patients in both groups and the retrospective patient selection, which leads to bias. These limitations may be overcome with future prospective trials.

In conclusion, the reversal of IFALD in NICU infants on combination LE containing FO is achieved by switching to Omegaven monotherapy. Our findings suggest that a change in the content of LEs used may have profound effects on the degree of liver dysfunction it may incite.

## Figures and Tables

**Table 1:** Clinical characteristics and outcomes of infants.

Case	GA (Weeks)	Birth Weight (g)	Diagnosis	Intestinal Anatomy	Sepsis Events	FO Initiated (DOL)	IFALD Resolved (DOL)	Duration of Treatment (Days)	Outcome
1	28 + 2	830	MRI	Jejunostomy	0	63	85	22	IFALD reversed
2	26 + 5	616	NEC	Jejunostomy	1	142	169	27	IFALD reversed
3	25 + 3	940	NEC	Jejunostomy	1	65	96	31	IFALD reversed
4	38 + 3	3680	Midgut volvulus	SBS *	0	30	69	39	IFALD reversed
5	28 + 3	960	NEC	Jejunostomy	0	38	71	33	IFALD reversed
6	28 + 5	620	MRI	Jejunostomy	0	60	104	44	IFALD reversed
7	27 + 6	1090	NEC	Jejunostomy	1	37	91	54	IFALD reversed
8	25 + 2	500	MRI	Jejunostomy	1	50	107	57	IFALD reversed
9	27 + 2	870	NEC	Jejunostomy	2	79	161	82	IFALD reversed
10	30 + 2	930	MRI	Jejunostomy	0	75	134	59	IFALD reversed
11	39 + 1	3920	MRI	Jejunostomy	0	33	59	26	Death (IFALD reversed) **
12	24 + 4	540	NEC	Jejunostomy	2	68	-	112	Death
13	26 + 0	855	NEC	Jejunostomy	1	164	-	38	Death
14	29 + 4	810	MRI	Jejunostomy	0	43	-	29	Death
15	26 + 0	370	NEC	Jejunostomy	2	56	-	61	Death

GA: gestational age; MRI: meconium-related ileus; NEC: necrotizing enterocolitis; IFALD: intestinal failure-associated liver disease; DOL: days of life. * 20 cm remnant small bowel and intact ileocecal valve. ** Death unrelated to IFALD or hepatic failure. All other deaths were IFALD-related.

**Table 2:** Biochemical data and growth of infants at the initiation and cessation of fish oil monotherapy.

Case	Total Bilirubin, mg/dL		Direct Bilirubin, mg/dL		AST, IU/L		ALT, IU/L		Albumin, g/dL		Triglyceride, mg/dL		Enteral Tolerance, %		Weight, Gram (Percentile)		Height, cm	
	A	B	A	B	A	B	A	B	A	B	A	B	A	B	A	B	A	B
1	6.8	20.1	5.5	15.0	119	200	74	126	2.6	3.2	132	134	5	50	1700 (<3)	1980 (<3)	40	41
2	3.9	1.8	2.6	1.4	87	55	68	53	3.1	3.3	99	59	0	0	3790 (<3)	3960 (<3)	45	49
3	7.7	27.0	7.0	23.5	46	762	41	229	2.4	3.3	136	208	0	60	1570 (25)	2800 (50)	41.5	44
4	4.0	1.6	3.0	1.3	101	46	137	70	3.0	3.5	88	111	5	60	4009 (50)	5350 (50)	53.6	59
5	4.6	2.9	4.1	2.4	74	214	71	674	3.2	3.6	319	102	0	45	1270 (<3)	1980 (<3)	39	40
6	19.1	4.8	16.0	4.0	44	100	29	155	2.5	4.3	95	201	25	50	1870 (<3)	2610 (<3)	40	41.5
7	9.9	1.1	8.7	1.3	336	30	124	87	2.1	4.2	122	94	0	50	1490 (25)	2170 (<3)	40	43
8	10.7	1.0	9.5	0.7	57	49	73	107	3.6	3.1	247	90	20	20	1080 (<3)	1950 (<3)	33.5	37
9	12.7	1.8	9.4	1.5	160	88	140	87	3.5	2.5	125	53	55	0	1750 (<3)	3430 (<3)	43	50.5
10	5.6	1.3	6.4	1.0	165	59	79	55	3.4	3.4	82	71	5	70	2910 (25)	4390 (50)	44.5	51
11	6.8	6.4	5.3	5.0	110	244	96	472	3.3	3.5	-	-	0	50	4220 (25)	4320 (50)	56	57
12	13.7	7.8	11.6	6.6	207	201	306	308	3.5	1.9	199	103	0	15	1090 (<3)	4160 (<3)	35	50.5
13	19.4	8.1	12.4	9.3	113	1132	86	328	5.2	2.5	147	95	10	0	2700 (<3)	3730 (<3)	49	50.5
14	5.2	13.9	5.1	12.0	110	180	75	53	3.4	2.0	167	97	0	0	1570 (<3)	2740 (50)	38	43
15	5.5	8.5	5.9	7.7	174	184	198	270	4.1	3.1	348	284	5	5	920 (<3)	1800 (<3)	33	34

A, initiation of fish oil monotherapy; B, cessation of fish oil monotherapy.

**Table 3:** Clinicopathologic characteristics and outcomes of infants with different IFALD management schemes.

	SMOF Reduction (N = 32)	FO Monotherapy (N = 15)	*p*-Value
Gestational age (mean)	27 + 3 weeks	28 + 3 weeks	0.16
Birth weight (mean, g)	1118.1	1168.7	0.89
Sex			
Male	23	11	0.72
Female	9	4	
Etiology			
NEC	18	8	0.12
MRI	4	6	
Duodenal atresia	4	0	
Jejunal atresia	3	0	
Midgut volvulus	1	1	
Omphalocele	1	0	
Age at PNALD diagnosis (mean, DOL)	43.5	66.9	0.19
Weight at PNALD diagnosis (mean, g)	1661.1	2129.3	0.16
Enteral tolerance at PNALD diagnosis (mean, %)	13.1	8.7	0.07
Biochemical parameters at PNALD diagnosis			
Direct bilirubin (mean, mg/dL)	4.1	7.4	0.04
AST (mean, IU/L)	66.3	126.9	0.11
ALT (mean, IU/L)	55.3	106.5	0.25
Albumin (mean, g/dL)	3.0	3.1	0.63
Outcome			
Survival	18	11	0.34
Death	14	4	

SMOF, SMOFlipid; FO, fish oil; NEC, necrotizing enterocolitis; MRI, meconium related ileus.
